# A Review: The Current In Vivo Models for the Discovery and Utility of New Anti-leishmanial Drugs Targeting Cutaneous Leishmaniasis

**DOI:** 10.1371/journal.pntd.0003889

**Published:** 2015-09-03

**Authors:** Emily Rose Mears, Farrokh Modabber, Robert Don, George E. Johnson

**Affiliations:** 1 College of Medicine, Swansea University, Swansea, United Kingdom; 2 School of Biological Sciences, The University of Auckland, Auckland, New Zealand; 3 Drugs for Neglected Diseases *initiative*, Geneva, Switzerland; 4 Center for Research and Training on Skin Diseases and Leprosy (CRTSDL), Tehran University Medical Sciences, Tehran, Iran; University of Antwerp, BELGIUM

## Abstract

The current in vivo models for the utility and discovery of new potential anti-leishmanial drugs targeting Cutaneous Leishmaniasis (CL) differ vastly in their immunological responses to the disease and clinical presentation of symptoms. Animal models that show similarities to the human form of CL after infection with *Leishmania* should be more representative as to the effect of the parasite within a human. Thus, these models are used to evaluate the efficacy of new anti-leishmanial compounds before human clinical trials. Current animal models aim to investigate (i) host–parasite interactions, (ii) pathogenesis, (iii) biochemical changes/pathways, (iv) in vivo maintenance of parasites, and (v) clinical evaluation of drug candidates. This review focuses on the trends of infection observed between *Leishmania* parasites, the predictability of different strains, and the determination of parasite load. These factors were used to investigate the overall effectiveness of the current animal models. The main aim was to assess the efficacy and limitations of the various CL models and their potential for drug discovery and evaluation. In conclusion, we found that the following models are the most suitable for the assessment of anti-leishmanial drugs: *L*. *major–*C57BL/6 mice (or–vervet monkey, or*–*rhesus monkeys), *L*. *tropica*–CsS-16 mice, *L*. *amazonensis–*CBA mice, *L*. *braziliensis*–golden hamster (or–rhesus monkey). We also provide in-depth guidance for which models are not suitable for these investigations.

## Introduction

Cutaneous Leishmaniasis (CL) is a poverty-associated disease that presents as lesions in the form of ulcers, nodules, papules, and plaques on exposed body parts (arms, legs, face). There are approximately 1 million new cases of CL each year; however, it is difficult to estimate the true incidence of leishmaniasis due to a lack of surveillance systems in remote areas and marginalized populations where the disease occurs, and because it is not a reportable disease within the health systems of many endemic countries. CL has a wide geographical range, with the majority of cases occurring in Afghanistan, Algeria, Colombia, Brazil, Iran, Syria, Ethiopia, Sudan, Costa Rica, and Peru. It is estimated that there are 20 species of *Leishmania* that cause CL, each with different epidemiology and natural history. Some species of *Leishmania* are more common than others, and their incidences vary widely amongst their geographical locations. The most common Old World species that cause CL are *L*. *tropica*, *L*. *aethiopica*, and *L*. *major* and the New World parasites include *L*. *mexicana*, *L*. *amazonensis*, *L*. *braziliensis*, *L*. *panamensis*, and *L*. *guyanensis* [1]. It must be noted that hybrid *Leishmania* have been isolated in nature and conjugation (transfer of genetic materials) has been demonstrated in the sandfly vector [2].

*Leishmania* infections typically originate from the bite of sandflies belonging to either *Phlebotomus* spp (Old World) or *Lutzomyia* spp (New World), which transmit the parasites into the skin of the host. The first sign of infection appears as a small erythema that develops at the site of the sandfly bite and advances within a few weeks to months. Lesions often become chronic after the initial erythema develops into a papule, then a nodule that may ulcerate over a period of two weeks to six months before becoming the characteristic lesion [3]. In strict CL, each lesion is representative of an independent fly bite, but, in rare cases, the disease can manifest as a disseminated disease (diffuse CL [DCL]) [4].

There is currently no satisfactory treatment for any form of CL and a need to develop short, safe, efficacious, affordable, and field-friendly treatments for the disease. The Drugs for Neglected Diseases initiative (DNDi) is a collaborative, patients’ needs-driven, nonprofit drug research and development (R&D) organization that is developing new treatments for neglected diseases, including drug discovery against CL. DNDi decided to focus on development of treatment for CL that is predominately caused by the *L*. *tropica* and *L*. *braziliensis* species. This is due to the severity and public health importance of these particular species; however, it is likely that these new treatments would be efficacious against CL caused by other parasites, too, as described in the 2007 consultative meeting to develop a strategy for treatment of cutaneous leishmaniasis [5]. Here, they suggest that these parasites will be targeted primarily because of the public health importance of the disease they produce. *L*. *tropica* is an anthroponotic parasite; therefore, treatment of cases is essential for controlling transmission. *L*. *braziliensis* is associated with mucosal leishmaniasis (ML) more often than other New World *Leishmania*, and one risk factor for developing ML is duration of infection. Hence, again, early treatment is important for control of ML. Furthermore, it was suggested that highly active drugs against *L*. *tropica* and *L*. *braziliensis* may be active against others (such as amphotericin-B). Decisions on how to treat CL are individualized. Treatment approach depends on a number of factors, including *Leishmania* spp. and/or geographic incidence as well as clinical characteristics and symptoms. For example, the oral agent miltefosine has FDA-approved indications that are limited to *L*. *braziliensis*, *L*. *panamensis*, and *L*. *guyanensis*.

Animal models can be used to evaluate drug candidates, with the goal of developing new drugs that offer a safe, effective, and shorter-course treatment for CL, as well as an acceptable safety profile. Therefore, it is essential to review the current animal models, which are highly diverse, and provide guidance on which models are the best for the discovery and utility of new anti-leishmanial drugs.

## Animal Models for Different Species of *Leishmania*

It must be noted that there is no validated animal model for CL, although these models are routinely used in several laboratories. Animal model validity refers to the similarity between the model and the human condition. Standardized animal models are used in laboratories that screen drug compounds against *Leishmania* strains to assure reproducibility. However, the predictive validity of the animal models is often low due to incomplete correlation between animal and human disease mechanisms. The philosophy is that if an agent was ineffective in these models, then no further development would be justified. Besides, some preliminary data on dose response and toxicity may be collected. Certain parameters must be considered when determining the similarity of an animal model to the human disease. These parameters include, but are not limited to, the clinical manifestations and presentations of the disease, disease pathogenesis, and the immunological responses that occur. For an animal model to be considered “validated” and used as part of preclinical development studies of an agent for therapy or prophylaxis, it must be similar to humans with respect to etiology, pathophysiology, symptomatology, and response to the therapeutic or prophylactic agents. For example, the BALB/c–*L*. *major* model is far from being a validated model for human CL based on the above criteria. Immunosuppression renders BALB/c mice resistant to *L*. *major* infection [6], whereas it has the opposite effect in humans. However, it is convenient, with easy read out using conventional methods. The inbred, resistant strains have many similarities with human disease, but don’t have the same pathological features and disease manifestations, such as secretions from the lesions (classically known as wet lesions), chronic persistent lesions, and the variety of disease presentation. Being inbred, these mice don’t show the variations in the presentation of the disease seen in humans; nevertheless, they are the most appropriate models available.

Ideally, outbred mice challenged by sandfly bites would be closer to human disease, particularly for vaccine studies, but it would require a large number of mice with complex endpoint evaluation (different disease manifestation) and a setup for sandfly challenge, which would be time-consuming and expensive and also require special expertise. It is therefore not practical for routine screening of drugs.

Many in vivo experimental models of both the New World and Old World CL have been developed for the study and testing of new compounds against the parasite, but few accurately reproduce the biological responses that would occur in humans. Inbred mice strains are most commonly used for experimental CL infections. However, other animal models, such as hamster and rat species (primary tests), dogs (secondary tests), and nonhuman primates (tertiary tests) have also been used. Examples of experimental CL involving dogs are lacking (though not unheard of [7–9]), and, as a result, this paper focuses solely on rodents and nonhuman primates as the potential models for drug evaluation. In vivo models for CL should aim to mimic the natural transmission of the disease—for example, the parasite load, presence of saliva, and site of inoculation—to enable accurate representation of disease progression. However, in many of the current models, this is not always the case.

The purpose of an animal model regarding CL is to investigate the parasitic cause of the human disease and use this information in drug studies to prevent increased risk to the human population. When exposed to the different *Leishmania* species, which each have individual pathogenic characteristics, the animal model should have pathological features and immunological responses that are similar or identical to humans. The model can then be used for the development and evaluation of new potential anti-leishmanial compounds and/or immunotherapy or therapeutic vaccines. There are many ethical issues that arise when using animal models, and thus their use should be avoided when possible. The three R’s of animal testing (Replacement, Reduction and Refinement), which were first described in 1955 by Russell and Burch [10], must always be considered as guiding principles for the more ethical use of in vivo models for the discovery of new drugs. The drug discovery algorithms for CL typically begin with the in vitro high throughput screening (HTS) during lead identification, which initially assesses the efficacy of large numbers of compounds against the parasites directly. The compounds that qualify as an in vitro hit are then assessed using in silico methods to determine their pharmacokinetic properties and toxicity during candidate identification. Assessing these properties via computer simulation has the potential to speed the rate of discovery without the need for expensive laboratory work. Drug discovery is a very innovative part of the drug development pipeline, with customised in vitro HTS and in silico packages, allowing for the identification of drugs with high on-target efficacy, low off-target toxicity, and favourable pharmacokinetics. Moreover, the in vitro results may potentially correlate with the in vivo studies [11] and the in silico ADME screening tools for pharmacokinetics and pharmacodynamics are gaining increasing accuracy and regulatory acceptance as well. Such weight is now given to these results that drug candidates with an unacceptable in silico and/or in vitro profile are excluded from further study by the drug developer. In vitro drug discovery has many advantages over in vivo testing, such as the very quick generation of results and the minute amount of test compound used. However, current drug screening methods in CL are laborious, especially when the intracellular amastigote model is used. In addition, the drug discovery test results of in vitro systems will always need to be verified in animals during drug development and before the human clinical trials of the compound begin. This is because the in vitro predictions for the compounds may not necessarily transfer to in vivo situations. Similarly, results of animal models may not be totally predictive of the response in humans, as evidenced by the low/lack of efficacy of allopurinol alone in humans, while it was shown to be active in vitro and in animal models [12–15].

It is also important to use recent isolates of *Leishmania* from the field for in vitro as well as in vivo tests. Some laboratories prefer to use old WHO reference organisms isolated in the 1960s–1970s for consistency and uniformity; however, *Leishmania* change over time and those circulating presently have different characteristics (Ana Rabello, personal communication). Changes have also been demonstrated in *L*. *donovani* parasites kept in culture (in vitro) compared to parasites freshly isolated out of rodents [16].

Nonhuman primate models are often used for the evaluation of potential anti-leishmanial compounds as a precursor to human clinical trials [17]. However, the results achieved from these nonhuman primate studies are often no better than those from the rodent models, and their use over rodent models may even be inferior [18]. The determination of parasite load within the animal model is also an important factor to consider when establishing the efficacy of a compound.

The requirement of an animal model capable of generating reproducible results is therefore crucial for the study of potential anti-leishmanial compounds. It is important to mention that, as of yet, the translation of results from preclinical to clinical is very poor, hence almost no compound has reached Phase III. With these factors in mind, it is important to determine the most effective animal model for CL drug discovery to help focus on robust systems and reduce the use of animals. Rodent models are most commonly used for studies of experimental CL infections, especially when studying the pathogenesis of disease or to test novel anti-leishmanial agents. This is because of the high availability of cellular markers and inbred, congenic, and now even transgenic mice, in addition to all other attributes that have made rodents a suitable animal for laboratory-based experiments. Mice in particular are susceptible to most strains and species of *Leishmania* in both the non-cure and self-cure models [19]. Much of the recent work towards the study of CL has been based on initial findings [20–22] deducing that different strains of mice, with differing phenotypes, vary in their susceptibility to different *Leishmania* species.

Some of the observations made in the rodent models of CL might not be similar or relevant to human hosts due to the distance in phylogeny. However, rodent models can provide a fast turnaround during the drug research program prior to testing in nonhuman primate models. Old World monkey species (macaques, baboons, mandrills, etc.) have the closest evolutionary relatedness to humans among the approachable nonhuman primate models [17]. Therefore, nonhuman primate models are generally used as the final experimental study of the safety and efficacy of drugs, with the hope that their relatedness to humans will confer a similar mechanism of CL infection and disease progression.

## Standardization of Inoculum

It is well known that promastigotes go through developmental stages (metacyclogenesis) during growth in culture. Therefore, the composition and size of inoculum as well as the method, route, and site of inoculation greatly influences the outcome of infection. It is therefore important that all these parameters are standardized for obtaining reproducible results.

During the course of in vitro growth, *Leishmania* undergo some essential changes (similar to that within sandfly vector) from highly dividing but non-infective form in log phase to non-dividing but highly infective metacyclic form in stationary phase [23]. Therefore, most laboratories harvest the parasite during the late stationary phase of growth and employ peanut agglutinin (PNA) to remove non-metacyclic parasites before inoculation. The size of inoculum is also important since, up to a certain level, higher doses produce larger lesions, which develop faster. Generally, 1 × 10^6^ metacyclic-enriched parasites were injected subcutaneously in a volume of 40μl. However, with the highly purified infective form of parasites, a few thousands of parasites are injected to simulate delivery by infected sandflies, which are estimated to be <5,000 [24]. The site of injection is also important for development of lesions. Footpads of mice are usually used for drug screening and provide easy measurement of lesion and parasite load at the lesion. The base of the tail of mice and the forehead of monkeys have also been used for vaccine studies. Another interesting site for mouse studies is the ear, which has also been used for inoculation by infected sandflies to simulate natural infection. Needle injections primarily deliver parasites subcutaneously, whereas sandfly introduces the parasite intradermally. The course of infections and the immune responses that ensue are different, primarily due to the type of inflammatory cells that appear soon after the introduction of parasites at the different sites [25]. Ideally, ear infection by infected sandflies would be preferable, particularly for vaccine development, since it is closer to natural infection in humans and has been shown to be different from needle challenge in vaccinated mice [24]. It should be noted that this method should be validated for humans first before making it a prerequisite for testing safe candidate vaccines in human clinical trials. However, this method requires availability of sandflies and consistent maintenance of infection rates. For drug screening, needle injection of a standard inoculum given subcutaneously in the food pad of mice would make it possible for many laboratories to screen drugs, albeit not simulating natural infection.

The composition of the inoculum with respect to the types of parasites (at various stages) should be determined and standardized. The PNA negative selection to enrich metacyclic *L*. *major* contains apoptotic parasites that can promote infection [26] and, ideally, their proportion in the inoculum should be known. The screening labs have their consistent method of growing the parasite, harvesting at a predetermined time, and preparing metacyclic-rich inocula within their own laboratories, in order to limit variation. However, comparison of results from different labs can be difficult because of the strain of parasite used, the age of the parasite since the isolation from the field, method of growth, separation of infective parasite, etc.

## Old World: *L*. *major*, *L*. *tropica*, and *L*. *aethiopica*

### *L*. *major*

It is a common misconception that the BALB/c–*L*. *major* model is the best for studies of immunology and drug evaluation regarding CL [27,28]. This misconception may be because the method of infection is highly reproducible and thus they are widely used as an in vivo model for CL drug discovery. Alternatively, the BALB/c–*L*. *major* has shown clinical [29] and immunological features closer to the human visceral disease [30–32]. All indicate the similarity of this model to human visceral leishmaniasis (VL) and not CL, except that a skin lesion is also produced. The majority of inbred mouse strains are resistant to infection by *L*. *major* (C3H/He, CBA, C57BL/6, 129Sv/Ev [33]) and develop local cutaneous lesions, which spontaneously resolve in 10–12 weeks. However, the BALB/c inbred strain contrasts to this and is well known for being highly susceptible to infection. The clinical features observed in this model are quite unlike the human disease [34] and there is much concern about its relevance, suggesting that alternative models for drug discovery should be developed. The BALB/c mice develop large skin ulcers, which expand and metastasize, leading to death [21]. The drug discovery algorithm by Grogl et al., 2013, describes the concise and rigorous stages of anti-leishmanial drug discovery using both the BALB/c–*L*. *major* and golden hamster–*L*. *panamesis* models [35]. However, the former BALB/c model has limitations when considering pathological similarities in humans. Alternatively, the use of *L*. *major* in a humanized mouse model has proven effective when predicting the possible side effects that may be observed in human clinical trials [36].

The *L*. *major* parasite is one of the most widely studied species in nonhuman primates. A study in 1987 by Githure et al. described that the vervet (*Chlorocebus pygerythrus*), Sykes (*Cercopithecus albogularis*), and baboon species are all equally susceptible to *L*. *major* infection, while bushbabies are resistant [37]. The vervet and Sykes monkeys, in particular, developed erythema and conspicuous nodules on the eyelids and ears, which rapidly become ulcerating lesions, before demonstrating a self-healing phenomenon within approximately 3 months. This is comparable to that observed in humans infected with *L*. *major* and thus the vervet and Sykes species are considered to be suitable models for drug trials against human CL. The *L*. *major* parasite has also been shown to infect the vervet monkey via natural infection in the wild [38], which enhances the model’s potential. Additionally, vervet, Sykes, and baboon species breed fairly well in captivity [39]. One of the most ethically acceptable models for drug and vaccine testing in nonhuman primates has been demonstrated in the *L*. *major*–rhesus monkey (*Macaca mulatta*) model [40,41]. Consistent with the human cases of CL caused by *L*. *major*, the parasite also causes a self-limiting CL of moderate severity, which resolves within three months, suggesting that this model could serve as a prelude to human vaccine trials. However, this model is not always feasible, given the enormous expense in conducting trials in monkeys. The *L*. *major–resistant* strains of mice are the most appropriate available model, because of similarities with human disease and because it is now more practical, with *L*. *major* carrying fluorescent genes or fluorogenic markers than before with standard methods of measuring efficacy. However, the *L*. *major*–rhesus model provides for evaluating immune response modifiers (to be used for immune-chemotherapy and prophylactic vaccines) that are not active in mice, i.e., CpG deoxyoligonucleotides with species specific sequences that can activate human and monkey cells in vitro. These have shown therapeutic and prophylactic properties in *L*. *major*–rhesus monkeys [42].

### *L*. *tropica*

*Leishmania tropica* is a major cause of CL in the Middle East and some areas of Africa. Infecting BALB/c mice with *L*. *tropica* results in non-ulcerating small nodules and delayed type hypersensitivity [43]. A susceptible murine model for infection by the *L*. *tropica* strain is needed, as there is insufficient knowledge on the pathogenicity of this parasite in mammalian hosts, due to its difficulty to establish infection in vivo. Previous rodent models for *L*. *tropica* infection have involved C57BL/6 and BALB/c mice [44], black rats (*Rattus rattus*) [45], and Sprague Dawley rats (*Rattus norvegicus* spp.) [46]. None of these models effectively represent the clinical manifestations of CL in humans. A suitable rodent model for *L*. *tropica* infection has only recently come to light in the study by Kobets et al., 2012, which examined the genetics and susceptibility of host response to *L*. *tropica* in BALB/c-c-STS/A (CcS/Dem) recombinant congenic (RC) mouse strains [47]. The CcS16 strain is more susceptible to *L*. *tropica* than BALB/c, carrying 12.5% of genes from the resistant parental strain STS/A and 87.5% of genes from the susceptible BALB/c strain. There is ongoing work to map the genes for susceptibility and response to *L*. *tropica* in CcS-16 [48]. This group deduced that the response to *L*. *tropica* is controlled by multiple genes with heterogenous effects, detecting eight loci in the CcS-16 strain that control host–parasite interaction. The identification of these complexities will now allow their functional basis to be elucidated and the subsequent detection of homologous processes in humans. The CcS-16 model therefore shows great potential for the evaluation of new anti-leishmanial drugs.

There are very few examples of nonhuman primate models infected with *L*. *tropica*. The *L*. *tropica* infection of rhesus monkey does not follow the same immune pattern of cutaneous infection as it usually does in humans and is not an appropriate host for the parasite [49]. Likewise, none of the four East African nonhuman primates studied by Githure et al., 1987, resulted in infection after challenge with *L*. *tropica* [37]. The current nonhuman primate *L*. *tropica* models will therefore be ineffective for drug discovery.

### *L*. *aethiopica*

There are very few murine models that show susceptibility to the *L*. *aethiopica* strain and the models that currently attempt to utilize it result in poor clinical outcomes that do not represent the human form of the disease. The pathogenesis of *L*. *aethiopica* infection has been demonstrated in BALB/c mice [50] whereby no ulceration of the lesion was observed and there were no overt clinical symptoms after 203 days of infection. Likewise, Childs et al., 1984, discovered that only slight swellings of the nose were seen across 12 different strains of inbred mice infected with *L*. *aethiopica*, suggesting that murine models show little susceptibility to the parasite [51]. The models do not represent the human form of the disease and thus are poor models for drug discovery.

Experimental infections of *L*. *aethiopica* in nonhuman primates have not been studied recently. A report by Hailu et al., 1995, evaluated the susceptibility of three species of monkey—vervet monkey (*Chlorocebus pygerythrus*), Gelada baboon (*Theropithecus gelada*), and Olive baboon (*Papio anubis*)—to the parasite [52]. Infections in all the vervet monkeys and half of the Gelada baboons produced recognizable lesions; however, no lesions were produced during infection in the Olive baboon. The maximum lesion size was observed among vervet monkeys and the scars that formed after complete healing of the ulcerating lesions were typical of the CL scars observed in humans. This species of monkey is, as of yet, the only nonhuman primate that is susceptible to infection with *L*. *aethiopica*. However, further experimental studies are needed to explore its potential as a nonhuman primate model of CL for the evaluation of new drugs, due to infection with *L*. *aethiopica*.

## New World: *L*. *mexicana*, *L*. *amazonensis*, *L*. *braziliensis*, *L*. *panamensis*, and *L*. *guyanensis*

### *L*. *mexicana*

Most inbred mice will display susceptibility to *L*. *mexicana* and its subspecies at high doses. However, when infected via a model that mimics natural transmission, the mice display marked differences in their ability to control parasite growth. Of the three strains of mice (BALB/c, C57BL/6, and CBA/J) that were subjected to a low parasite dose of 10^3^ *L*. *mexicana* promastigotes inoculated via the intra-dermal route of infection, BALB/c proved to be the most susceptible, despite all mice strains previously showing an equal susceptibility when inoculated with high dose (10^6^) *L*. *mexicana* promastigotes subcutaneously [53].

The recent model by Sosa-Bibiano et al., 2012, made use of local mammalian fauna, the Yucatan deer mouse (*Peromyscus yucatanicus*), as a model host to study the development of *L*. *mexicana* infection with the aim of mimicking natural infection conditions [54]. The typical human patient will develop a localized cutaneous nodular ulcerated lesion when infected by *L*. *mexicana*, and this symptom presented itself in seven of 36 (19.44%) *P*. *yucatanicus*. Additionally, the scars that resulted in *P*. *yucatanicus* were round or oval-shaped with smooth surfaces, and showed a depression along with hypopigmentation, much like the healed lesions in humans with CL. However, the absence of these local clinical signs was observed in 29 of the 36 animals (80.55%). Although these former results support the utility of *P*. *yucatanicus* as a novel experimental model to study LCL caused by *L*. *mexicana*, more work is needed to utilize the model for drug discovery.

There is no recent work on the use of *L*. *mexicana* as the parasite for infection in nonhuman primate models.

### *L*. *amazonensis*

Infection by *L*. *amazonensis* may lead to the development of diffuse CL in a few human patients, which is characterized by nodular lesions. Recently, *L*. *amazonensis*-infected female BALB/c mice were used to evaluate the efficacy of different CL treatments [55]. The animals were inoculated with 10^7^ promastigotes through subcutaneous injections at the base of the tail. Despite some treatments showing promising results in vivo, limitations of the study included the use of an extremely susceptible animal model and the lack of an extended period to follow up on healing lesions. Many additional mouse strains are susceptible to *L*. *amazonensis*, in particular, the CBA strain, which develops severe lesions in comparison to C57BL/6, presenting small chronic lesions [56]. An advantage of the C57BL/6 mouse is the large number of gene knockout lines in the C57BL/6 background that can be used to address important questions such as the contribution of a specific immune response to drug efficacy. In the study by Charret et al., 2013, the CBA strain was chosen over the BALB/c strain due to its susceptibility to infection by *L*. *amazonensis*, developing cutaneous lesions without intense parasite dissemination [18]. It was also noted from Lemos de Souza, et al., 2000, that CBA mice infected with *L*. *amazonensis* produce increased levels of IL-4 and IL-10, whilst CBA mice infected with *L*. *major* produced IFN-γ and IL-10 [57]. In humans, IL-4 is associated with disease development and thus the similarity in immune response suggests the CBA strain has potential in replicating the human form of CL. This CBA–*L*. *amazonensis* model shows potential for use as a method for the development of new anti-leishmanial drugs.

Dose-dependent lesion development was observed in both the rhesus monkey (*Macaca mulatta*) and tufted capuchin (*Cebus apella*) infected by *L*. *amazonensis* [58,59]. Amaral et al., 1996, infected rhesus monkeys with *L*. *amazonensis* and found that animals inoculated with 10^8^ promastigotes consistently resulted in establishment of infection, including earlier onset and/or larger size of lesions [58]. The cutaneous lesions of infected monkeys shared the pathologic features of CL in humans: amastigotes with mononuclear infiltrate of macrophages, lymphocytes, and plasma cells, with the formation of tuberculoid-type granulomas. However, the DCL that could occur in humans was not observed in the animals during the time course of experiments and it is likely that the anergic form would not have developed according to the level of immune responsiveness found.

Garcez et al., 2002, successfully used the *C*. *apella* monkey for experimental studies of American CL and as a model for vaccine trials [59]. An intermediary dose of 2 × 10^6^ promastigotes was sufficient to induce consistent cutaneous lesions in these monkeys. This dose is extremely large in comparison with the dose injected by a vector in natural infection that varies from 10 to 1,000 promastigotes [60]. However, under this high dose, all animals presented nodular lesion development. *C*. *apella* monkeys only developed an erythematous nodule during primary infection that varied in size and persistence, curing spontaneously, unlike humans infected by *L*. *amazonensis* that typically developed characteristic ulcerations that do not typically self-cure. Although this *L*. *amazonensis*–*C*. *apella* model is considered to be a good model for vaccine trials, it is perhaps not as good as other models (such as the *L*. *amazonensis*–rhesus monkey), due to many aspects of infection that are dissimilar to the same disease in humans.

### *L*. *braziliensis*

Less experimental work has been conducted involving the infection of rodents with *L*. *braziliensis*, despite its wide distribution and burden in South America. *L*. *braziliensis* does not grow easily in vitro [61]. Most mouse strains are resistant to infection by *L*. *braziliensis* due to the inability of this species to inhibit the Th1 immune response [62]. Mouse strains such as C3H/HeJ, C57BL/6J, and CBA/CaJ show no evidence of infection by *L*. *braziliensis*, whereas strains AKR/J and CBA/J showed only a slight and transient swelling of the infected tissue, when parasites were inoculated in the snout [51]. Additionally, the *L*. *braziliensis* parasite has previously been shown not to produce severe or lasting cutaneous lesions in the BALB/c mouse strain [20], which has even been shown to kill the *L*. *braziliensis* parasite after infection and is therefore highly resistant [62]. However, a few reports have employed a BALB/c model in vaccination studies that successfully obtained chronic ulcerated lesions after infection by *L*. *braziliensis* promastigotes [63,64]. The addition of salivary gland exudates promotes infection in mice due to the presence of highly active vasodilators. Some of these proteins (LJM11) are now being used to generate blocking antibodies/cellular response in vaccine studies to reduce infection and lesion formation [65].

The only model which shows true potential for the evaluation of potential drugs targeting *L*. *braziliensis* is the golden hamster (*Mesocricetus auratus*). Hamsters are commonly used for strains of *Leishmania* that show low susceptibility to mice (*L*. *Viannia* subspecies), and disease progression can be monitored over longer periods of time due to the chronic nature of the disease in the rodent [14]. Gomes-Silva et al., 2013, demonstrated that, after infection with *L*. *braziliensis*, all hamsters developed cutaneous lesions very similar to those observed in humans during the observational period (110 days), with no spontaneous healing [66]. Although this particular hamster model shows predictable disease evolution, other models using different isolates and/or protocols have not shown reproducibility, especially in contrast to other well-established mouse/*Leishmania* models such as the BALB/c–*L*. *major* model. Nonetheless, the golden hamster could be recommended for clinical vaccine or therapeutic studies against *L*. *braziliensis* due to the fact that the chronic state of the disease closely resembles non-healing human CL and possibly reproduces some of the immunopathological aspects of the human disease. Unfortunately, the wider use of hamsters is still limited for CL drug discovery due to the lack of available reagents—such as antibodies to cell markers and cytokines specific to the CL response [67]—in addition to the higher doses of drugs required to dose hamsters compared to mice.

The *L*. *braziliensis*–rhesus model induced self-healing CL in which the T cell-mediated inflammatory response effectively promoted parasite clearance and granuloma resolution, with the lesional granulomas being remarkably similar to those seen in humans infected with the *L*. *braziliensis* pathogen [68]. A similar study by de-Campos et al., 2010, also confirms that the model can be used to elucidate the regulatory mechanisms that may render granulomas inadequate for fighting intracellular pathogens [69]. Further study of the various immune suppression mechanisms that induce granulomas in *L*. *braziliensis*-infected rhesus monkeys may reveal new opportunities for therapeutic control of the disease. The marmoset (*Callithrix penicillata*) has also been shown to develop a cutaneous lesion at the point of inoculation by *L*. *braziliensis* [70]. In this experiment, all but three of the 14 marmosets developed this lesion three to nine weeks after infection, and were characterized by the appearance of subcutaneous nodules containing parasites. The model may serve as a useful experimental model for the study of CL in the future, but for now, further work is needed.

### *L*. *panamensis*

The initial study by Neal and Hale, 1983, demonstrated that the BALB/c mouse strain is susceptible to high inoculums of *L*. *panamensis* promastigotes [20]. Subsequent work by Castilho et al., 2010, utilized this knowledge to develop a chronic infection model for *L*. *panamensis* using numbers of parasites that would more closely approximate infection in nature [71]. 5 × 10^4^ *L*. *panamensis* promastigotes generated chronic lesions in BALB/c mice with persistent parasites at the site of the infection for over one year. They also discovered that the immune response of the mouse resembles that found for *L*. *panamensis*-infected patients with chronic and recurrent lesions. Thus, the murine model should be useful in the understanding of the pathology of the human disease and may eventually be used as a model to develop methods for the treatment and prevention of CL caused by *L*. *panamensis* parasites. The golden hamster has also been shown to be susceptible to *L*. *panamensis* infection [72] but the cutaneous metastatic lesions vary considerably between animals and thus the model is unreliable. There is little research into *L*. *panamesis*-infected nonhuman primate models, however, one early study was reported using aotus monkey for evaluation of allopurinol [13].

### *L*. *guyanensis*

There are very few examples of *L*. *guyanensis*–rodent animal models. However, the golden hamster has been shown to provide an experimental model for most *L*. *Viannia* species, including *L*. *guyanensis* [72,73]. The extent of the model’s usefulness is limited, however, and not representative of the human disease. Very little research has been conducted into *L*. *guyanensis*–nonhuman primate models of infection and hence their use for the evaluation of potential anti-leishmanial compounds remains unclear.

It is important to note that there are many experimental variables that can be used to modulate disease outcome. These variables involve methods in the administration of the disease and affect phenotypes, such as modular or ulcerative lesions and fast or slow development of disease. These variables, including parasite dose [74], site of inoculation [75], co-injection with sandfly saliva [76], and temperature [77], have all been shown to affect disease outcome in animal models, thus demonstrating the importance of mimicking the natural route of infection in all animal models.

## Continuous Monitoring of the Parasite Load in the In Vivo Models, Using Reporter Molecules

Leishmaniasis affects many millions of people worldwide and hence there is an ever-growing need for a rapid screening assay to allow the high throughput screening of potential anti-leishmanial compounds. The current classical methods of in vivo parasite load determination are laborious, time consuming, and do no support automation. Furthermore, methods such as lesion size measurements are not always indicative of parasite killing due to immunological responses such as inflammation, which might also contribute to lesion size. Previously, methods required the animals to be euthanized at various times after infection. Quantification was then achieved by measuring the parasite load either by microscopy, limiting dilution assay, or q-PCR amplification of the parasite DNA. As Calvo-Alvarez et al. wrote, “Furthermore, this approach has some important limitations that must be overcome: (i) postmortem analysis of animals makes it impossible to track the space/time progression of the pathogen within the hosts; (ii) spread of the pathogen to unexpected anatomic sites can remain undetected; (iii) in order to achieve precise and relevant data, it is necessary to kill large numbers of animals” [78]. The measurements of lesion size and footpad and ear thickness are all common methods used to quantify the progression of the disease and hence the parasite number [35,66]. However, these methods require an incubation period of about two weeks before any visual observations of infection can be made. Thus, it is important to devise a way of detecting parasite numbers in the earlier stages of infection, before a visual phenotype is observed. More recently, the in vivo imaging system (IVIS) has been developed to allow the detection of measurable signals associated with cells in living organisms [79]. It is extremely important to quantify the parasite load present in an in vivo model in a live host, in order to determine the infectivity of the *Leishmania* parasite and/or to measure the efficacy of a potential anti-leishmanial drug.

Many reporter molecules have been devised for the quantification of protozoan parasites and each one comes with different degrees of sensitivity, advantages, and disadvantages. Reporter genes must encode a gene product that has a readily measurable phenotype that must also be distinguishable over a phenotypic background [80]. As described by Dube et al., 2009, “The ideal reporter gene should be (i) absent from the host; (ii) inert (and should not affect the physiology of the parasite cell); and (iii) should represent a simple, sensitive and inexpensive assay for quantification of reporter expression” [81]. Continuous monitoring of parasite load in vivo is an ideal way to monitor drug treatment outcome.

### β-galactosidase

β-galactosidase is widely used as an intracellular reporter gene and is the product of the bacterial *lacZ* gene. The primary advantages of using β-gal as a reporter gene is its simplicity, low cost and sensitivity. Depending on the substrate used, it offers a number of detection options [81]. The reporter molecule cannot be applied directly in vivo to observe the parasite load and progression in real time analyses, but β-galactosidase-positive *L*. *amazonensis* parasites were easily detected in mouse tissue as blue spots after staining with X-Gal [82]. Nevertheless, a considerable drawback to this method is the large size of the β-gal-expressed protein (116kDa) and its endogenous expression within most mammalian cell lines (macrophages), resulting in a false positive readout for parasite growth [81]. For this reason, the molecule is inaccurate for the quantification of parasite load, especially when determining the efficacy of a potential drug.

### β-lactamase

Unlike β-galactosidase, the reporter molecule β-lactamase shows no endogenous expression by mammalian cells and hence proves to be an excellent choice for protozoan reporter gene assays [83,84]. Betalactamases are a family of bacterial enzymes that cleave penicillins and cephalosporins [81]. The method is rapid, sensitive, and nonradioactive. Bukner and Wilson, 2005, engineered strains of *L*. *major* and *L*. *amazonensis* expressing the β-lactamase gene in an episomal vector [83]. Their method facilitated the relatively high throughput screening of the intracellular stages of the *Leishmania* strains against chemical libraries with potential anti-leishmanial activity. However, its utility was still limited as it was necessary to keep strong selective pressure over the parasite in culture to avoid the possible elimination of the parasite’s episomal construct. This colorimetric assay also requires the lysis of cells, and repeated measurements are not always possible. The β-lactamase reporter gene system devised by Mandal et al., 2009, uses transgenic parasites to optimize their utility in amastigote–macrophage drug screening assays, and is a vast improvement of the 2005 Buckner and Wilson model [84]. Currently, the β-lactamase reporter gene has not been applied directly in vivo.

### Luciferase

The gene expressing luciferase has been cloned from several different species, the firefly luciferase being the most commonly used form. Luciferase catalyses the oxidation of luciferin in the presence of Mg^2+^ and ATP into oxyluciferin, which produces a short flash of light directly proportional to the number of luciferase enzyme molecules. Thus, it provides a rapid indication of the transcriptional activity of the gene [81]. Roy et al., 2000, was able to visualise transfectant *L*. *major* strains stably expressing the firefly luciferase gene in vivo to monitor disease progression in real time analyses [85]. Likewise, a recent paper by Thalhofer et al., 2010, describes the IVIS technology which enabled the noninvasive visualization of luciferase-expressing *Leishmania* spp. parasites in living anesthetized BALB/c mice in real-time [79]. This technique allowed bio-imaging at the highest efficiency with the absence of background activity by host cells. A similar study was conducted by Lang et al., 2005 [86]. The IVIS technique has also been utilized to assess drug therapy (amphotericin B) on *L*. *amazonensis* infection in the skin of BALB/c mice [87]. This model enabled successful visualization of the decrease in parasite load after treatment with AmB in the mouse model. However, the method also comes with its disadvantages. The methodology is limited by the requirement of the substrate luciferin, which must be administered intravenously or intraperitoneally for the detection of the luciferase activity. This makes the technique expensive [81]. Additionally, the luminescence produced is unstable and dependent upon the metabolic activity of the cells, which have been transfected with luciferase.

### Green Fluorescent Protein

All of the above mentioned reporter genes require exogenous substrates, which limit their use and introduce an extra step in the detection of reporter expression. Using fluorescence for determining parasite load has many advantages over other reporter molecules. The fluorescent proteins work intrinsically, which allows real-time analysis of the molecular events in living cells. Additionally, fluorescent reporters do not require specific substrates and the fluorescence emitted is very stable over time. The use of fluorescence allows greater simplicity, easier kinetic monitoring, low cost, and enhanced biosafety [88], and thus has great potential for in vitro HTS of potential drug candidates. Green Fluorescent Protein (GFP) is the most commonly used fluorescent reporter molecule. It has been used over a large number of species for in vitro drug screening due to its flexibility, sensitivity, and the additional possibility of automization of the screening process using flow cytometry or fluorometry [88]. Variants of the GFP have been developed, which have optimal properties for high-throughput screening. These variants have different emission spectra and can fluoresce at wavelengths longer than that of native GFP, as seen in the enhanced green fluorescent protein (EGFP) [89]. More recently, the stable transfection of the EGFP reporter has been found suitable for in vivo as well as in vitro infection studies, showing potential use in drug screening assays in vivo, as there are no considerable variations in virulence or infectivity due to the genetic manipulation of the parasites [89,90]. Infrared fluorescence has recently been utilised as a tool for in vitro, ex vivo, and in vivo models of visceral leishmaniasis [91]. Here, in vivo noninvasive imaging of the visceral infection in BALB/c mice was achieved using transgenic fluorescent parasites. It is possible that this method may be transferred to studies of CL, opening up the possibility of testing vast amounts of potential drug compounds.

The introduction of the fluorescent reporter gene into the *Leishmania* parasite can be either transgenic or episomal. The use of an episomal vector carrying a given gene is limited for two reasons: (1) Gene expression is extremely heterogeneous in populations of transfected parasites due to the wide variation in the copy number of plasmids per cell. (2) The host loses the plasmid in the absence of an antibiotic resistance marker. These problems can be overcome by the integration of the reporter DNA into the parasite genome, thus creating a permanent transfectant [92]. Expression of GFP as an episomal transgene is useful for drug screening in either promastigotes or intracellular amastigotes in vitro (by flow cytometry [93]) and in vivo [94], but fluorescence in those parasites was very heterogeneous due to variation in the number of copies of the transgene. For the reasons described, it is more practical to consider transgenically introduced reporter genes in order to efficiently determine the parasite load. The GFP reporter system still has its disadvantages. The excitation maximum is close to ultraviolet (UV) range, which can cause damage to living cells. Additionally, the GFP model is not sensitive enough to enable a precise and reliable microplate screening in vitro, and, consequently, an in-depth flow cytometric analysis is required.

EGFP-labelled *L*. *major* transfectants have been detected in both the amastigote and promastigote stages in the footpad lesions of BALB/c mice [89]. The study showed an in vivo correlation between parasite fluorescence and footpad thickness, suggesting that the method could be applied as a semi-quantitative parameter for parasite load. The paper also suggests that the model could be utilized for in vivo real-time whole body fluorescence imaging and eventually be applied to different animal species such as hamsters and dogs. Pulido et al., 2012, used Bolhassini’s system to describe the first New World *Leishmania* strain (*L*. *panamensis*) to stably express GFP transgenically [90]. Their in vivo model uses the golden hamster, which they described as the best animal model for CL (based on work by Hommel et al., 1995 [95]), infected with *L*. *panamensis* plR3(-)eGFP. Fluorescence was homogenous and persisted for long periods of time. Promastigotes taken from the infected hamster were used to infect and produce induration in the hamster nose, indicating that the virulence of recombinant strains is not affected by genetic manipulation. Transfected cells remained as a homogenous and unique population.

A recent paper by Calvo-Alvarez et al., 2012, suggests that fluorescent reporter molecules that emit light in the red spectrum prove excellent candidates for in vivo drug screening assays [78]. The group used mCherry as their red reporter molecule, which was considered the best choice for their studies based on improved photostability and suitability for intravital imaging. The transgenic *L*. *major* LV39c5 + mCherry strain was used to infect BALB/c mice. Lesion size and fluorescence intensity showed good correlation. As the authors wrote, “It is remarkable that a weak but measurable fluorescence signal from infected hind limbs was detectable two weeks prior to visible and measurable injury took place in all dose groups” [78], and the in vivo model was subsequently applied to a vaccination study. Fluorescence was lower in the vaccinated group than the control by 80%. These results were reproducible, thus indicating that the model can be considered a highly suitable system for drug discovery. The mCherry fluorescent protein system is highly sensitive—being able to detect 10^4^ amastigotes isolated from a lesion—and shows possibilities for automation and miniaturization, reducing both cost and time.

Another recent paper utilises two reporter proteins: EGFP and luciferase combined [96]. They generated stable transgenic *L*. *major* expressing fused EGFP and firefly luciferase simultaneously, thus increasing the experimental sensitivity. These signals from the parasite were measurable within a mammalian host (BALB/c mice) and promastigotes. Their results concluded that both quantitative reporter genes could be used to detect parasite load in vitro and in vivo and demonstrated that firefly luciferase was 10-fold more sensitive than EGFP in promastigote stage. This model also has exciting potential for use in drug discovery.

## Discussion

To summarize, animal models that show the greatest potential for the evaluation of new potential anti-leishmanial drugs have not been certified, and this review goes some way in clarifying our understanding of the predictive validity and representativeness of the models for human disease. Currently, the BALB/c–*L*. *major* model is one of the most widely used. The idea of using BALB/c mice for drug studies is based on the notion that if the BALB/c mice can be protected or cured from the parasite disease, then so can the resistant mouse strains. However, using this model will possibly result in the inability to detect potentially useful compounds due to the stringencies and peculiarities associated with it. The risk of overlooking such compounds is too great, and thus, the model has limitations. Despite this, it should be noted that the choice of a rodent model strongly depends on the research question that is being addressed. The use of BALB/c–*L*. *major* can be justified in drug testing, as it is reproducible and generally accepted to be a rigorous test. CL progresses quickly in this non-healing model; hence, when a lesion size and parasite load reduction is obtained, it is likely to lead to reduction in other self-cure models. The in vivo model should be considered solely as a predictive tool in a complete set of experiments involving in vitro and additional in vivo work in which each experiment answers a different defined question, as demonstrated by Grogl et al., 2013.

It is usually difficult to refute a dogma. The BALB/c–*L*. *major* model has been used for testing drugs for decades [97,98], primarily because of extreme sensitivity of BALB/c to *L*. *major* infection and ease of outcome evaluation. It is now clear that the outcome of the infection is directly influenced by the immune responses of the host. It is also known that *L*. *major* infection in BALB/c triggers a strong Th-2 response to leishmanial antigens, leading to rapid lesion growth and generalized infection and, eventually, death. Hence, as mentioned elsewhere in the text, immune-suppressive agents, which control this Th-2 response, reduce the lesion size and parasite load. We would like to caution that reduction in lesion size and parasite burden may not be due only to the anti-parasitic activity of the agents being tested but also their effects on the immune responses. As of yet, the most comprehensive rodent models are the *L*. *tropica*–CsS-16 [48], *L*. *amazonensis*–CBA [18], and *L*. *braziliensis*–golden hamster [66], which each show promise for drug discovery (Table 1).

**Table 1:** The recommendation of various animal models with *Leishmania* parasite–animal models for discovery of new anti-leishmanial drugs, based on the evidence and interpretation of the studies reviewed in this paper.

*Leishmania* Parasite	Animal Model										
	Rodent models							Nonhuman primate models			
	BALB/c	CBA	C57BL/6	CsS-16	Humanized mice	golden hamster (*Mesocricetus auratus*)	Yucatan deer mouse (*Peromyscus yucatanicus*)	vervet monkey (*Chlorocebus pygerythrus*)	Sykes monkey (*Cercopithecus albogularis*)	rhesus monkey (*Macaca mulatta*)	tufted Capuchin (*Cebus apella*)
*L*. *major*	Th2/Th1. Visceral disease and death	Th1>Th2. Self-cure	+++, Th1>Th2. Self-cure		++, Develop cellular components of the human immune system; T, B and NK cells.			+++, Self-healing lesion. IFn-g production by circulating cells does not correlate with cure.	++, Self-healing lesions.	+++, Self-healing immune responses similar to humans	
*L*. *tropica*	No lesion. Slow growth		++, Slow developing lesion	+++, Females develop large skin lesions. CCL3/CCL5							
*L*. *aethiopica*	No growth							++, Self-healing lesions			
*L*. *mexicana*	++, Large nonhealing lesions						++, Single small lesion				
*L*. *amazonensis*	++, Th2, Lesions	+++, Develop severe lesions	Th1. Small chronic lesions							++, Self-healing lesions.	++, Self-healing lesions, Th1/Th2
*L*. *braziliensis*	++, Small self-healing lesions. Th1 response		Small nonulcerated lesions			+++, Nonhealing ulcerated lesions				+++, Self-healing lesions. Th1 response	
*L*. *panamenisis*	++, Self-healing lesions. Th1/Th2										
*L*. *guyanensis*						++, Metastatic lesions					

**+++** = Strong evidence for recommendation

++ = More research needed before recommendation

Blank boxes confer to lack of sufficient information/data

Many *Leishmania* species are ineffective at producing human-like immune and clinical responses in nonhuman primate models, or else have not been widely studied (*L*. *tropica*, *L*. *mexicana*, *L*. *panamensis*, and *L*. *guyanensis*). The *L*. *major*–vervets model is one that well represents the human form of disease and has the additional advantage of the animal breeding relatively well in captivity. The vervet monkey has also been shown to produce recognizable lesions after infection with *L*. *aethiopica*, but more research is needed to evaluate the models’ potential for drug discovery. The *L*. *major–*rhesus model also shows consistency with human cases of CL and has been used in vaccine evaluation. Both the rhesus monkey and *Cepus apella* develop clinical symptoms after infection with *L*. *amazonensis*, however, the former model is more representative of the human CL disease and thus may be better for drug trials. The rhesus monkey also develops characteristic lesional granulomas remarkably similar to those seen in humans infected with *L*. *braziliensis*. Therefore, the *L*. *major*–vervets (37), *L*. *major*–rhesus [40,41], and *L*. *braziliensis*–rhesus [68,69] models show the greatest potential for the evaluation of new potential anti-leishmanial drugs.

The use of nonhuman primate models for the evaluation of potential anti-leishmanial compounds is often used as a precursor to human clinical trials. However, the results achieved from these nonhuman primate studies are often inferior to those that use rodent models. Nonhuman primates should only be used as a model for drug evaluation if rodent models are incapable of reproducing reliable results. As of January 1, 2013, Directive 2010/63/EU replaced Directive 86/609, stating that the use of nonhuman primates can only be used under strict circumstances, for example, for the benefit of human beings when it involves life-threatening or debilitating diseases. Nonhuman primates must only be used if there is scientific justification to the effect that the purpose of the procedure cannot be achieved by the use of species other than nonhuman primates. Their use should not be sanctioned during the early stages of research and is more appropriate for toxicology testing at the preclinical stage of development. Often, the rodent models are far better at assessing the efficacy of a drug, and some reproduce aspects of human infection to a far greater extent than the nonhuman primates. When considering in vivo models for drug discovery purposes, it should be noted that only small fractions of drugs are available. Relatively restricted amounts of drug are used in mice, being smaller than hamsters. Nonhuman primate models require much higher doses that are often not available at early stages. Animal models that show potential for studies of drug evaluation should have obtained the disease through a method that mimics natural infection and, additionally, should present similar clinical symptoms as are observed in humans. It is also important to choose the right animal model for the *Leishmania* species, since it has been demonstrated in multiple clinical trials that the efficacy of a given compound varies depending on the species of *Leishmania* causing the infection. These factors should be considered although it is acknowledged that such parameters are not always feasible. From this analysis, we conclude that *L*. *major*–vervet, *L*. *major*–rhesus, *L*. *tropica*–CsS-16, *L*. *amazonensis*–CBA, *L*. *braziliensis*–golden, or *L*. *braziliensis*–rhesus are the best models for CL drug discovery. However, the use of these nonhuman primate models should only be used under extreme cases whereby they conform to the new directive and are scientifically justified.

Neither β-galactosidase nor β-lactamase are effective reporter molecules for in vivo drug discovery. However, their use in vitro is widely used due to their simplicity and low cost. The use of the firefly luciferase reporter molecule in transgenic *Leishmania* species shows exciting results in its use for drug evaluation due to its high sensitivity and use in real-time analyses; however, the process is costly due to the need for the substrate luciferin. Alternatively, fluorescent reporter molecules can be used to determine parasite load and have the advantage that they do not require the use of exogenous substrates. Fluorescent proteins work intrinsically, allowing for the real-time analysis of the molecular events in living cells. Although the GFP and its variants have been used to quantify *Leishmania* parasites in in vivo models previously, there are concerns that the excitation maxima of these proteins are close to the UV range, causing damage to living cells. The recent mCherry + *L*. *major* model has been shown effective in a vaccination study in BALB/c mice and has greater sensitivity over the more commonly used GFP models. Additionally, the combined use of EGFP and firefly luciferase in the *L*. *major*–BALB/c model demonstrates a highly sensitive method for parasite quantification that has potential for drug evaluation.

It is important to emphasize the extra valuable information we can obtain using reporter molecules as tools for calculation of parasite load when compared to conventional methods. The conventional methods used to quantify parasite load, such as the direct measurement of parasite via microscopy or q-PCR amplification of parasite DNA, are not only laborious and time consuming, but also require the euthanasia of large numbers of animals. In addition, these methods cannot detect the spread of pathogens to unexpected anatomic sites or track their space/time progression. In contrast, reporter molecules provide a readily measurable phenotype that allows the continuous monitoring of parasite load in vivo. These tools are highly sensitive and often show potential for automation resulting in high-throughput quantification, especially when combined with computational methods.

Current animal models for the utility and discovery of new potential anti-leishmanial drugs differ vastly in their immunological response to disease and clinical presentation of symptoms. After the initial HTS of the drugs in vitro, animal models are used to ensure correlation of drug results in vivo before human clinical trials begin. Infected animal models with similarities to the human form of CL should be more representative when considering the effectiveness of a compound within a human. Models with dissimilar clinical presentations should be avoided. The accurate quantification of parasite load and, thus, effect of a drug must then be determined through the use of reliable quantification methods as described. It should also be noted that the overall curative effects of anti-leishmanial drugs in vivo depend not only on antiparasitic activities of the agents but also on immune responses of the host. Hence, understanding the immunopathology of the animal model is important when selecting agents for clinical development.

In conclusion, we found that the following models are the most suitable for the assessment of anti-leishmanial drugs: *L*. *major*–C57BL/6 mice or–vervet monkey, *L*. *major*–rhesus, *L*. *tropica*–CsS-16, *L*. *amazonensis*–CBA, *L*. *braziliensis*–golden or *L*. *braziliensis*–rhesus. We also found that the other models investigated here are not currently supported. The guidance provided in this report should help when considering a CL animal model for drug discovery, and we suggest that further work should focus on these recommended models.

**Key Learning Points**

- It is important to choose the right animal model for the *Leishmania* species, since it has been demonstrated in multiple clinical trials that the efficacy of a given compound varies depending on the species of *Leishmania* causing the infection.
- In many cases, rodent models are far better at assessing the efficacy of a drug, and some reproduce aspects of human infection to a far greater extent than nonhuman primates.
- Quantification of the parasite load present in an in vivo model is important to determine the infectivity of the *Leishmania* parasite and/or to measure the efficacy of a potential anti-leishmanial drug.
- We conclude that *L*. *major–*vervet (or–rhesus), *L*. *tropica–*CsS-16, *L*. *amazonensis–*CBA, and *L*. *braziliensis–*golden (or–rhesus) are the best models for CL drug discovery.

**Top Five Papers**

1. Reithinger R, Dujardin JC, Louzir H, Pirmez C, Alexander B, Brooker S. Cutaneous leishmaniasis. Lancet Infect Dis. 2007 Sep;7(9):581–96.
2. Nasseri M, Modabber FZ. Generalized infection and lack of delayed hypersensitivity in BALB/c mice infected with Leishmania tropica major. Infect Immun. 1979 Nov;26(2):611–4.
3. Neal RA, Hale C. A comparative study of susceptibility of inbred and outbred mouse strains compared with hamsters to infection with New World cutaneous leishmaniases. Parasitology. 1983 Aug; 87(Pt 1):7–13.
4. Dube A, Gupta R, Singh N. Reporter genes facilitating discovery of drugs targeting protozoan parasites. Trends Parasitol. 2009 Sep;25(9):432–9.
5. Calvo-Álvarez E, Guerrero NA, Alvarez-Velilla R, Prada CF, Requena JM, Punzón C. Appraisal of a Leishmania major strain stably expressing mCherry fluorescent protein for both in vitro and in vivo studies of potential drugs and vaccine against cutaneous leishmaniasis. PLoS Negl Trop Dis. 2012;6(11):e1927.
